# Intra-Colonial Viral Infections in Western Honey Bees (*Apis Mellifera*)

**DOI:** 10.3390/microorganisms9051087

**Published:** 2021-05-18

**Authors:** Loreley Castelli, María Laura Genchi García, Anne Dalmon, Daniela Arredondo, Karina Antúnez, Ciro Invernizzi, Francisco José Reynaldi, Yves Le Conte, Alexis Beaurepaire

**Affiliations:** 1 Laboratorio de Microbiología y Salud de las Abejas, Departamento de Microbiología, Instituto de Investigaciones Biológicas Clemente Estable, Montevideo 11600, Uruguay; castelli.loreley@gmail.com (L.C.); danielarpapiol@gmail.com (D.A.); kantunez03@gmail.com (K.A.); 2 Consejo Nacional de Investigaciones Científicas y Técnicas (CONICET), La Plata 1900, Buenos Aires, Argentina; ml.genchigarcia@gmail.com (M.L.G.G.); freynaldi@yahoo.com (F.J.R.); 3 Instituto Multidisciplinario de Biología Celular (IMBICE), La Plata 1900, Buenos Aires, Argentina; 4 Laboratorio de Virología, Facultad de Ciencias Veterinarias, Universidad Nacional de La Plata (LAVIR-FCV-UNLP), La Plata 1900, Buenos Aires, Argentina; 5 Abeilles et Environnement, INRAE, 84000 Avignon, France; anne.dalmon@inrae.fr (A.D.); yves.le-conte@inrae.fr (Y.L.C.); 6 Sección Etología, Facultad de Ciencias, Montevideo 11400, Uruguay; cirobee@gmail.com; 7 Institute of Bee Health, University of Bern, 3003 Bern, Switzerland

**Keywords:** evolutionary biology, host–pathogen interactions, population genetics, viruses, pollinators

## Abstract

RNA viruses play a significant role in the current high losses of pollinators. Although many studies have focused on the epidemiology of western honey bee (*Apis mellifera*) viruses at the colony level, the dynamics of virus infection within colonies remains poorly explored. In this study, the two main variants of the ubiquitous honey bee virus DWV as well as three major honey bee viruses (SBV, ABPV and BQCV) were analyzed from *Varroa-destructor*-parasitized pupae. More precisely, RT-qPCR was used to quantify and compare virus genome copies across honey bee pupae at the individual and subfamily levels (i.e., patrilines, sharing the same mother queen but with different drones as fathers). Additionally, virus genome copies were compared in cells parasitized by reproducing and non-reproducing mite foundresses to assess the role of this vector. Only DWV was detected in the samples, and the two variants of this virus significantly differed when comparing the sampling period, colonies and patrilines. Moreover, DWV-A and DWV-B exhibited different infection patterns, reflecting contrasting dynamics. Altogether, these results provide new insight into honey bee diseases and stress the need for more studies about the mechanisms of intra-colonial disease variation in social insects.

## 1. Introduction

RNA viruses stand amongst the most diverse and widespread disease-causing agents [1,2]. In these pathogens, genetic variation is enhanced by the accumulation of mutations during replication and their rearrangement by genetic recombination [3,4]. This high mutation rate allows RNA viruses to generate collections of closely related viral genomes around one or more master genotypes [5,6,7]. Virus transmission can occur in two different ways: horizontally, i.e., among individuals of the same generation (directly through contact between individuals, contaminated colony food or food sources, or indirectly through biological vectors), and vertically, i.e., from parents to offspring [8]. Notably, the horizontal transmission of viruses is widespread in social species, where barriers between individuals are significantly reduced due to diverse factors, such as high densities and high rates of interaction [9,10].

RNA viruses play a major role in the pollinator losses currently observed across the globe [11,12]. Many studies have focused on their impact on the health of the western honey bee (*Apis mellifera*) [13,14]. In this species, the impact of RNA viruses is greatly enhanced by the invasive ectoparasite mite *Varroa destructor*. In fact, while feeding on honey bees, Varroa can act as a vector for highly virulent viruses [15,16]. *V. destructor* also influences the immune status of its host, triggering uncontrolled replication through immunosuppression [17,18]. As a consequence, after the introduction of this parasite into novel ecosystems, honey bee viral landscapes significantly change [19,20,21,22]. Among the 70 different viruses that have been characterized in honey bees, a great proportion were reported to be transmitted by *V. destructor*, including the ubiquitous *Deformed wing virus* (DWV), *Acute bee paralysis virus* (ABPV), *Kashmir bee virus* (KBV) and *Israeli acute bee paralysis virus* (IAPV) (reviewed in Beaurepaire et al. [23]). Due to the multiple routes of infection, resulting in frequent co-infections [24], *Black queen cell virus* (BQCV) and *Sacbrood virus* (SBV) are also often detected in *A. mellifera* colonies [25,26].

In honey bee epidemiology, the colony is often considered the basic unit, suggesting that viral loads are uniform among individuals from the same hive. However, due to their polyandrous mating system, *A. mellifera* colonies display genetic heterogeneity across individuals emerging from the same queen, which may be of high relevance when studying pathogens [27]. Thus, within a colony, workers can share the same parents, or their fathers can differ, resulting in half-sister groups, also called ‘subfamilies’ or ‘patrilines’. According to Sherman et al. [28], queens benefit from mating with more than one male to reduce the probability of having an offspring mainly composed of genotypes susceptible to a pathogen. Polyandry may indeed provide beneficial non-additive genetic interactions among subfamilies by capturing rare alleles that regulate resistance to pathogens and parasites in a population. Consequently, genetic variation within colonies may increase the chance of surviving diseases [29,30]. This theory predicts that different subfamilies found in a colony express distinct resistance levels to pathogens with which they co-evolved [31]. However, empirical studies testing this theoretical prediction remain scarce. 

The aim of this study was to analyze the patterns of infection of some of the major honey bee RNA viruses at the intra-colonial level. This was achieved by comparing virus infection levels between individual *A. mellifera* pupae of known patrilines across distinct periods and under different *V. destructor* infestation statuses. The results documented here reveal new insights into the ecology of honey bee viruses and stress the need to further consider intra-colonial heterogeneity in disease infection for social insect research.

## 2. Materials and Methods

Colonies of *A. mellifera* were sampled in the INRAE institute’s apiary (Avignon, France) from the end of August to the end of October 2018 to study the relationships between mite infestation and worker subfamilies (see [32]). In brief, worker brood cells were screened in colonies not treated against *V. destructor* for over a year before running the experiments. Reproductive characteristics of *V. destructor* mites were analyzed by opening brood cells and examining their content (pupae age, recapping behavior, foundress number and all other Varroa stages).

The phenotyped individuals were dissected directly upon sampling. DNA from 486 collected pupae (purple eye stage) was isolated using a hind leg per individual according to the Chelex extraction protocol described by Walsh et al. [33]. The patrilines of the pupae were reconstructed using microsatellite markers, and the relationship between mite infestation traits and brood genotypes was tested. The rest of the collected pupae were stored on dry ice during sampling and transferred at −80 °C for further analysis directly after the phenotyping.

In the current study, individuals belonging to the most prevalent subfamilies in the three colonies genotyped from Beaurepaire et al. [32] (mean = 9.82 pupae per subfamilies) were analyzed. These samples, all infected by one reproducing or non-reproducing foundress *V. destructor*, originated from three colonies with a total of 36 samples per colony. Accordingly, the 36 selected pupae per colony belonged to 3–4 distinct subfamilies (Table 1). These 108 individuals were used to study patterns of infection with four honey bee viruses, including two DWV variants (DWV-A, DWV-B, ABPV, SBV and BQCV) (Table 2).

Each pupa was crushed in 800 µL of QIAzol (Qiagen, Courtaboeuf, France) containing a 9 mm silver bead using a TissueLyser (Qiagen) at 30 Hz for 30 s with 30 s intervals and repeated four times, according to the protocol described in Dalmon et al. [34]. The tube was centrifuged for 1 min at 12,000× *g* and 4 °C. RNA extraction was performed using 0.5 mL of crude extract of each pupa. An RNeasy Plus Universal mini kit (Qiagen) was used according to the manufacturer’s protocol.

The RNA was quantified using a Nanodrop^®^ spectrophotometer (Thermo Scientific, Illkirch, France) and normalized at 500 ng/µL. Reverse transcription was performed with the high-capacity RNA to cDNA kit (Applied Biosystems, Saint Aubin, France) using 1 µg RNA in 20 µL total reaction volume containing random primers according to the manufacturer’s protocol.

The number of virus genome copies of each virus was determined by quantitative PCR using a StepOne-Plus Real-Time PCR System (Applied Biosystems^®^, Thermo Scientific, Illkirch, France). Three microliters of ten-fold diluted cDNA were added to 5 µL of SYBR Green master mix (Applied Biosystems). Five reactions were conducted independently, each containing 10 µM of forward and reverse virus and variant-specific primers [35,36] (Table 2). Amplification was performed using the following program: 10 min at 95 °C, 40 cycles of 15 s at 95 °C, 1 min at 60 °C and a melting curve was generated from 60 to 95 °C. Absolute reference to the corresponding synthetic DNA fragments (Eurofins Genomics, Ebersberg, Germany, Table S1) was used to calculate the mean number of virus genome copies per pupa. Detection thresholds were set up at Cq = 31. Quantification was replicated twice per sample and included negative controls for reverse transcription and qPCR. All samples except negative controls tested positive at least for one virus.

**Table 2:** Set of primers for quantitative PCR. The name, primer sequence, fragment size of the PCR product, annealing temperature (Tm) and primer references are provided. Corresponding synthetic DNA fragments used for quantification are listed in Table S1.

Name	Sequence (5′–3′)	Size (bp)	Tm	Ref.
DWVnew-F1	TACTAGTGCTGGTTTTCCTTT	156	79.5 °C	[37]
DWVA-R1	CTCATTAACTGTGTCGTTGAT			
DWVB-R1	CTCATTAACTGAGTTGTTGTC			
ABPV-F6548	TCATACCTGCCGATCAAG	197	82.1 °C	[36]
KIABPV-B6707	CTGAATAATACTGTGCGTATC			
SBV-qF3164	TTGGAACTACGCATTCTCTG	335	78.5 °C	[36]
SBV-qB3461	CTCTAACCTCGCATCAAC			
BQCV-qF7893	AGTGGCGGAGATGTATGC	294	81.6 °C	[36]
BQCV-qB8150	GGAGGTGAAGTGGCTATATC			

To assess the intra-colonial patterns of viral infections across the sampled pupae, variations of honey bee viruses across *V. destructor* infestation levels and reproduction type, and among brood age categories, sampling periods, colonies and subfamilies, were compared. All statistical tests described below were conducted with R v. 4.0.4 [37], and the levels of significance used were corrected for multiple comparisons using the Bonferroni method to adjust *p*-values when required.

Given that pupae of different ages (i.e., 5 to 9 days old) were collected during the sampling, virus genome copies were compared between the different age categories using a linear regression to test whether virus infection slopes differed significantly from zero. These tests were non-significant, thereby enabling the grouping of samples of different ages for subsequent analyses (Figure S1). As the samples were collected during two distinct sampling periods (I: end of August until mid-September and II: October), levels of virus infection at these two periods were compared using Wilcoxon–Mann–Whitney tests for each colony independently. In cases of significant time differences, samples were grouped by period for subsequent analyses. One colony (colony B) was removed from this temporal analysis as only three samples were collected in this colony during the second sampling period (Table 1).

Differences of virus genome copies among the three colonies and among the patrilines within each colony were tested using non-parametric tests: Wilcoxon–Mann–Whitney tests were used when comparing two groups, Kruskal–Wallis tests were used to compare >2 groups together and Dunn tests were used in the case of significant differences for subsequent pairwise comparisons. Notably, only groups with at least six samples were considered for these statistical analyses.

Finally, the number of virus genome copies of the colonies was compared to the mite infestation level in the brood in the same matching period. Moreover, as all pupae chosen for the experiment were infested with a single foundress mite, successfully reproducing or otherwise, additional Wilcoxon–Mann–Whitney tests were applied to assess the effect of mite reproduction on the virus genome copies.

## 3. Results

### 3.1. Overall Virus Quantification

**Figure 1:**
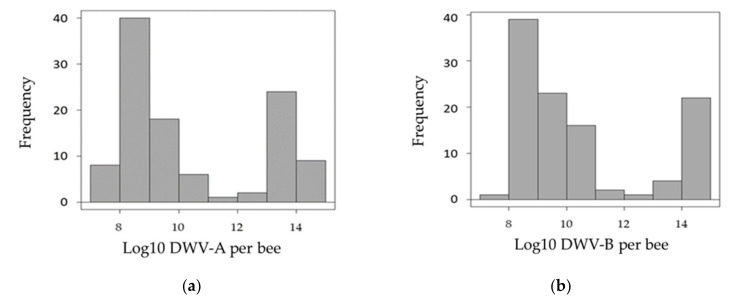
Overall frequency of DWV-A and DWV-B. The distribution of infections with DWV variants (Log10) is shown as the proportion of pupae with between 10^7^ and 10^15^ DWV genome copies/nymph. (**a**) Histogram for DWV-A; (**b**) histogram for DWV-B.

**Figure 2:**
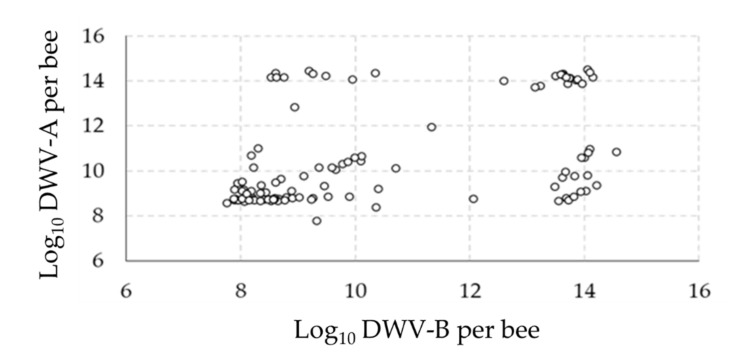
Relations between DWV variants. The relationships between the DWV variant (DWV-A and DWV-B) levels in the samples are represented by plotting the infection with both variants for each pupa.

Of the four viruses investigated (DWV, ABPV, SBV and BQCV), only one (DWV) was found to be over the detection thresholds (Appendix A). All samples were infected by the two variants of this virus (DWV-A and DWV-B). Preliminary observations of the distribution of DWV variants in the pupae showed that most samples were either moderately (<10^10^ genome copies/pupa) or strongly (>10^12^ genome copies/pupa) infected (Figure 1). Interestingly, when observing the number of virus genome copies with the two variants, four groups could be distinguished: (i) a high number of genome copies of both variants, (ii) a low number of genome copies of both variants, (iii) a high number of genome copies of DWV-A but a low number of genome copies of DWV-B and (iv) a low number of genome copies of DWV-A but a high number of genome copies of DWV-B (Figure 2).

### 3.2. Virus Genome Copies across Time and Colonies

**Figure 3:**
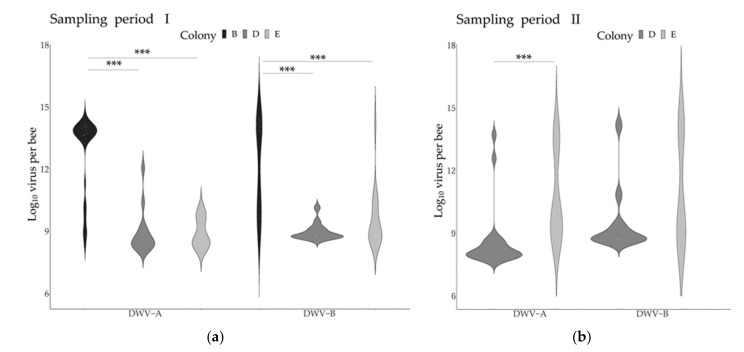
DWV variant genome copies across colonies and sampling periods. The graphs represent the levels of DWV-A and DWV-B across colonies and sampling periods. (**a**): Sampling period I (end of August to mid-September); (**b**): Sampling period II (October). The asterisks represent statistical significance after Bonferroni corrections (Period I: Kruskal–Wallis and Dunn tests; Period II: Wilcoxon–Mann–Whitney tests). Only groups with a sample size ≥ 14 were included (sample sizes: Period I: colony B: 33; Colony D: 14; Colony E: 22. Period II: Colony D: 18; Colony E: 18). Asterisks reflect significance levels.

The comparison of the DWV variants genome copies across the two collection periods revealed contrasting temporal patterns in the two colonies tested. Significant increases in DWV-A were detected in both colonies, while the levels of DWV-B did not change significantly over time (Table 3, Figure 3). Given these temporal patterns, the different periods were considered independently for the subsequent analyses for both variants. Comparing the virus genome copies across the different pupae age stages (day 5 to 8 post-capping) did not reveal any significant differences, although slight decreases in virus genome copies during pupal development could be observed (Figure S1).

When analyzing the DWV-A and DWV-B infection levels across the colonies, significant differences were found during the first sampling period between colony B and the other two colonies (Figure 3).

### 3.3. Virus Genome Copies across Subfamilies

**Figure 4:**
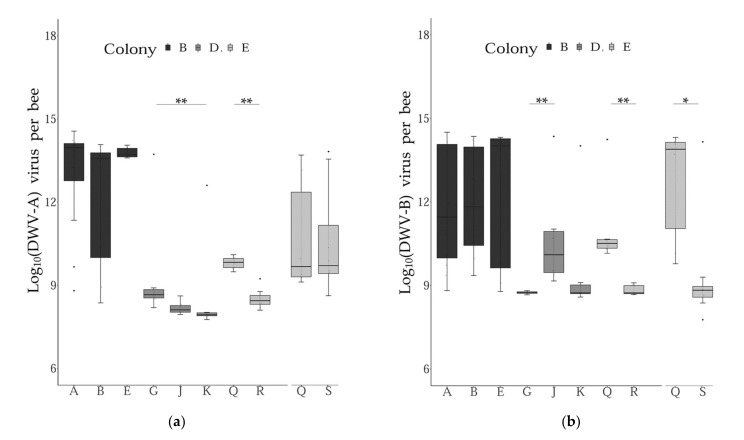
DWV variant genome copies across patrilines. Boxplots representing the level of infection across pupae grouped by patrilines within each colony and sampling periods (Col B: period I; Col D: period II; Col E period I and II). The asterisks indicate levels of significance of the non-parametric tests conducted (Wilcoxon–Mann–Whitney or Dunn tests corrected with Bonferroni). (**a**): DWV-A genome copies; (**b**): DWV-B genome copies. Error bars represent 95% confidence intervals.

Comparing the levels of DWV variants between the different subfamilies within colonies and sampling periods revealed some significant differences across the groups tested. More precisely, significant differences were found when comparing two and three patrilines for their infection levels with DWV-A and DWV-B, respectively (Table S2, Figure 4). Interestingly, these results varied for the two DWV variants investigated, i.e., some patrilines were differentially infected by one variant, but not with the other.

### 3.4. Virus Genome Copies and Mite Infestation

When comparing the level of mite infestations of the colonies with the number of virus genome copies of DWV-A and DWV-B, similar patterns were found across the colonies and time periods. More precisely, virus genome copies of both variants appeared to be positively correlated to levels of mite infestations in the brood for two colonies (B and E), but not for the third colony (D) (Figure S2). Finally, comparing pupae infected by a cell with a reproducing vs. a non-reproducing mite did not reveal any statistically significant differences between the two groups (Wilcoxon–Mann–Whitney tests, all *p* > 0.05, Table S3).

## 4. Discussion

In this study, patterns of infections by honey bee viruses were investigated within *A. mellifera* colonies. Although four common viruses were screened for, only one (DWV) was detected. Investigating two variants of this virus (DWV-A and DWV-B) revealed novel insights into the biology of this major bee pathogen by unravelling new features of its epidemiology across time, colonies, subfamilies and individuals.

To date, the natural infection of honey bee viruses has been principally studied in adults, and very rarely in the brood [24]. In the present study, 108 five to nine-day-old pupae were sampled and analyzed for four major honey bee viruses. However, three out of the four viruses analyzed were below the detection thresholds. Notably, low infection with BQCV, ABPV and SBV in *A. mellifera* pupae sampled in fall in France was also documented in another study [38]. Given the variety of factors (e.g., time, colonies and patrilines) playing a significant role in DWV infection patterns documented here, it is important to conduct follow-up studies including higher sample sizes per colony to perform more powerful statistical analyses (i.e., models) in order to determine the contribution of each factor individually, as well as interactions between variables. Another important aspect to consider is that, although the DWV-A and DWV-B are known to recombine readily [34,39], recombinants between these two variants could not be analyzed with the methods used here. Nevertheless, the results presented in this study bring several new insights into the dynamics of the two major DWV variants.

First, the analysis of DWV variant infection levels overall, as well as between the analyzed colonies, revealed that the pupae were either moderately (<10^10^ genome copies/pupae) or strongly (>10^12^ genome copies/pupae) infected. Furthermore, investigations of the relationships between the two variants of DWV at the individual level revealed four distinct groups of pupae: high infections with both variants, moderate infections with both variants and both cases of high infection with one strain and moderate infection with the other. Such puzzling patterns have been described in previous studies, with DWV-A and DWV-B showing a bimodal distribution between symptomatic and asymptomatic adult honey bees [40] and delimitations between groups of about 10^6–8^ DWV-A copies/bee and 10^9–10^ DWV-B copies/bee [41,42]. Here, the virus genome copies data indicate that the majority of the pupae were infected to levels corresponding to future symptomatic adults [43]. Such high virus infections may result from the fact that all cells analyzed were parasitized by one *V. destructor* foundress and from the absence of treatments against this DWV-vector for more than one year in the colonies prior to sampling.

Two of the colonies analyzed here (colonies D and E) originated from a Varroa-surviving population [44], while the third one (colony B) was from a local susceptible stock that requires frequent mite treatment to survive. This might explain the higher number of genome copies of DWV variants observed in colony B compared to the other two colonies. Notably, some populations of naturally surviving *A. mellifera* colonies (i.e., that do not require Varroa treatment) display lower virus infection levels in the brood [45]. However, more colonies from both the surviving and susceptible stocks used here should be tested to confirm that this surviving *A. mellifera* population displays tolerance or resistance to virus infections.

As DWV variant levels differed across colonies, the temporal dynamics of virus genome copies were analyzed at the colony level. This analysis revealed a variation of temporal infection between the two DWV variants, with significant differences in virus genome copies detected between the two sampling periods when comparing the levels of DWV-A, though not of DWV-B. As similar patterns were detected when comparing the genome copies of the two variants to the colony-level brood infestation with *V. destructor*, the vector was probably not responsible for the differences observed. This suggests that other factors governing the dynamics of the two DWV variants might explain the temporal differences observed when comparing DWV-A and DWV-B over the sampling periods. For instance, differential mortality associated with the two variants [46,47] might have played a role, since more lethal pathogens are generally less likely to transmit [8].

The results of this study also showed that patterns of DWV-A and DWV-B infections can differ significantly across honey bee pupae, even when these hosts belong to the same colony and share the same environment (i.e., the pupae were all infested by a *V. destructor* foundress and were collected on the same frame). Moreover, when individuals were grouped by subfamilies, significant differences in DWV-A and/or DWV-B genome copies were found. Notably, the significant variation of virus genome copies across subfamilies is in line with previous work showing differential infection in honey bee subfamilies with two other pathogens: *Paenibacillus larvae* [48] and *Ascosphaera apis* [49]. Three non-exclusive hypotheses come to mind when trying to explain the results obtained here. First, DWV, as well as the other viruses that were screened for in this study (ABPV, SBV and BQCV), can be transmitted venereally [50,51,52]. Thus, it is possible that the different levels of the two DWV variants observed were caused by venereal transmission, i.e., from differentially infected drones to their offspring. Additionally, differences in DWV genome copies may have also resulted from interactions with other microorganisms, such as pathogens [53,54] or the host’s gut microbiota [55]. Finally, differential resilience towards virus infection exhibited by the pupae subfamilies could explain these results [56,57]. In this case, resistance mechanisms inherited by the pupae from their sires may have caused the patterns observed. A recent study documenting the ‘suppressed *in ovo* virus infection’ showed that virus resistance has a strong genetic component in *A. mellifera* [58], thereby supporting the latter hypothesis. From this statement, we can infer that virus infections may vary in the different pupae subfamilies within a colony due to the differential expression of resistance traits across pupae originating from different sires. However, although significant differences were found when comparing subfamilies, important variations were observed within patrilines as well. This suggests that other factors might play a role in determining DWV infection levels in *A. mellifera* pupae and stresses the need for more studies to identify the source of this variation.

In this study, significant variations of infection between patrilines were observed for DWV-A and DWV-B. DWV-B is known to have proliferated in Europe more recently than DWV-A [47,59]. Despite the differences of exposure of the host to the variants, the variability across honey bee subfamilies documented here suggests that the interactions between the two DWV variants and their hosts have both led to the evolution of adaptations in *A. mellifera* and/or the viruses. However, the fact that the differences of DWV-A and DWV-B infection were not consistent across patrilines suggests that distinct biological mechanisms might be at play in the interactions among *A. mellifera, V. destructor* and these two variants.

## 5. Conclusions

The results documented here show novel patterns of intra-colonial variation in virus infection that reveal an underestimated complexity in the spatiotemporal dynamics of infection by honey bee viruses. These findings stress that intra-colonial heterogeneity should be more carefully considered when conducting projects about honey bee diseases, as well as other social insects with similar life-history traits (i.e., polyandry or polygyny). Colonies are often considered an epidemiological unit, but this might fail to account for the variability described here, leading to erroneous conclusions. In this study, potential co-evolutionary interactions between hosts and virus genotypes may have caused the differential interactions between the two variants of DWV and the individual and subfamily levels of infections. If parental genetic components are indeed responsible for variations in virus infection levels, new avenues for the breeding and selection of more disease-resilient honey bee colonies can be explored. This could contribute to ensuring more sustainable beekeeping globally, as well as providing new knowledge on host–pathogen interactions and the importance of genetic diversity in disease epidemiology.

## Figures and Tables

**Table 1:** Number of samples and subfamilies per *A. mellifera* colony screened for RNA viruses. The colony of origin (Colony); subfamily as described in Beaurepaire et al. [32] (Subfamily = patriline); sample size per subfamily (N); per period (N_Period_: I for end of August to mid-September and II for October) and per foundress reproduction groups (N_Reproduction_: R for reproducing foundress and NR for non-reproducing foundress).

Colony	Subfamily	N	N_Period_	N_Reproduction_
B	A	12	I: 12; II: 0	R: 5; NR: 7
	B	10	I: 8; II: 2	R: 2; NR: 8
	C	6	I: 5; II: 1	R: 4; NR: 2
	E	8	I: 8; II: 0	R: 8; NR: 0
D	G	7	I: 1; II: 6	R: 5; NR: 2
	J	12	I: 6; II: 6	R: 9; NR: 3
	K	10	I: 4; II: 6	R: 8; NR: 2
	L	7	I: 3; II: 4	R: 5; NR: 2
E	Q	12	I: 6; II: 6	R: 10; NR: 2
	R	12	I: 8; II: 4	R: 10; NR: 2
	S	12	I: 4; II: 8	R: 10; NR: 2

**Table 3:** Statistical analysis of temporal changes in DWV genome copies. The table indicates the result of Wilcoxon–Mann–Whitney tests conducted to study the differences of DWV, DWV-A and DWV-B between the two sampling periods (I: end of August to mid-September and II: mid- to late October). Asterisks reflect significance levels. NA: not available (sample sizes too low).

Colony	DWV-A	DWV-B
Col B	NA	NA
Col D	W = 231.5, *p* = 0.012 *	W = 147, *p* = 0.8328
Col E	W = 68, *p* = 0.003 **	W = 116, *p* = 0.1496

## Data Availability

All additional data can be obtained from the corresponding author upon reasonable request.

## Supplementary Materials

The following are available online at https://www.mdpi.com/article/10.3390/microorganisms9051087/s1, Figure S1: Comparison of virus levels across brood stages; Figure S2: Viral genome copies and mite infestation levels, Table S1: Standard Gene Information; Table S2: Result of statistical tests comparing the levels of DWV across patrilines; Table S3: Results of the Wilcoxon–Mann–Whitney tests to compare cells with reproducing and non-reproducing mites.

## Appendix A

Full dataset including cq values for each sample analyzed.
