# Supplementary material for: Intra-Colonial Viral Infections in Western Honey Bees (Apis Mellifera)
Source: Microorganisms. 2021 May 18;9(5):1087. doi: 10.3390/microorganisms9051087 (PMC8158351; doi:10.3390/microorganisms9051087)
Supplement: Supplementary file 1 [file microorganisms-09-01087-s001.zip › microorganisms-1187569-supplementary/Castelli et al. 2021 - Supplementary Material.pdf]

microorganisms

Article

Intra-colonial viral infections in Western honey bees (*Apis mellifera*)

Loreley Castelli[1] +, María Laura Genchi García[2,3,4] +, Anne Dalmon[5], Daniela Arredondo[1], Karina Antunez[1], Ciro Invernizzi[6], Francisco José Reynaldi[4,7], Yves Le Conte[5], Alexis Beaurepaire[5,8] *

Supplementary Material

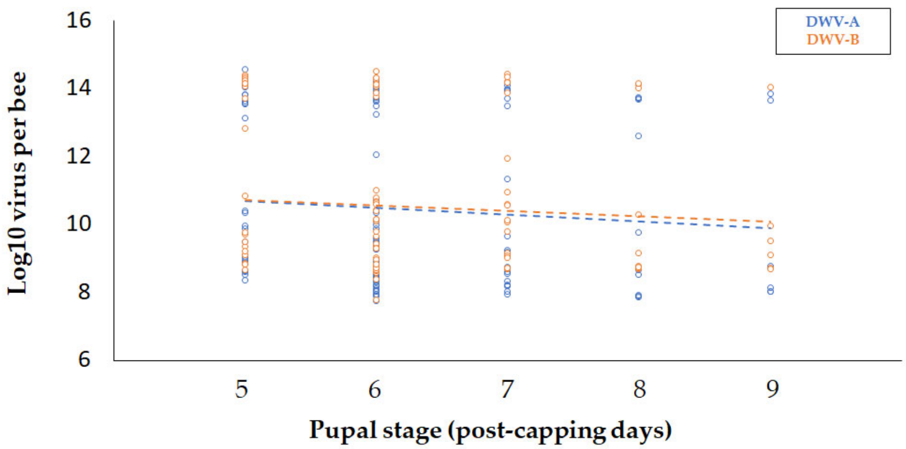

Figure S1 - Comparison of virus levels across brood stages

DWV strain titers of individual pupae across different brood stages are shown. The dotted lines represent regression lines. The statistical tests were non-significant for the two strains (linear regressions, $p > 0.05$).

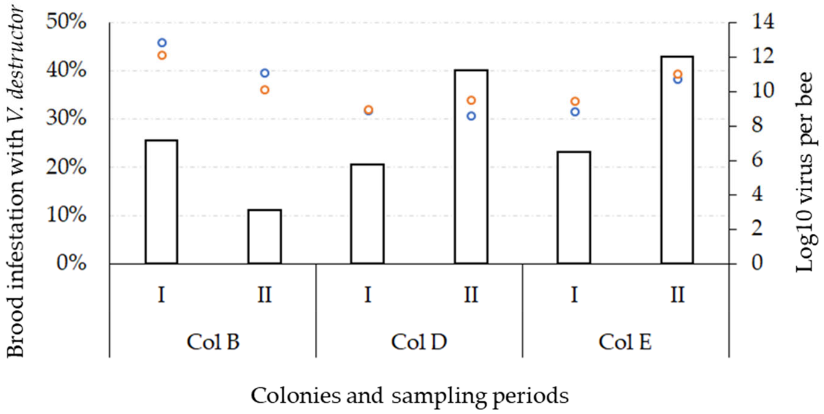

Figure S2 - Viral loads and mite infestation levels

Graph representing the *V. destructor* brood infestation levels at the time of sampling (columns) and the mean log10 of DWV-A (blue dots) and DWV-B (orange dots) virus genome copies (right axis).

Table S1 - Standard Gene Information

Standard genes were synthesized by Eurofins Genomics, Vector Backbone pEX-A128, Antibiotic Selection: Ampicillin, Cloning via Type IIS restriction enzymes.

Virus (size)	Sequence
DWV-A (156nt)	TACTAGTGCTGGTTTTCCTTTGTCTTCATTAAAGCCACCTGGAACATCAGGTAA<br>GCGATGGTTGTTTGATATTGAGCTACAAGACTCGGGATGTTATCTTTTGCGAG<br>GAATGCGTCCCGAACTCGAGATTCAATTATCAACGACACAGTTAATGAG
DWV-B (156nt)	TACTAGTGCTGGTTTTCCTTTATCTTCATTAAAACCGCCAGGCTCTTCTGGTAA<br>GCGATGGTTGTTTGATATTGAATTACAAGATTCAGGATGTTATCTTTTGAGAG<br>GGATGAGACCTGAACTTGAGATACAGTTGACAACAACTCAGTTAATGAG
BQCV (294nt)	AGTGGCGGAGATGTATGCGCTTTATCGAGGAGGAGTTCGAGTTAAAGTTGTT<br>ACTGAGAAGGGTGTGGATTTCGTCAGAGCTACCGTTAGTCCTCAACAGACTT<br>ACGGCAGTGATGTCGCTCCTACTACTCATATCAGTACTCCTTTGGCAATAGAA<br>CAAATACCTATAAAGGGAGTCGCAGAGTTCCAAATACCGTACTATGCTCCAT<br>GTTTGTCATCTTCGTTTAGAGCGAATTCGGAAACATTTTACTATAGTTCAGGTC<br>GGAATAATCTCGATATAGCCACTTCACCTCC
ABPV (197nt)	TCATACCTGCCGATCAAGAAACAAATACTTCCAACGTACATAATACGCAACT<br>CGCGTCGACCTCTGAAGAAAACTCAGTTGAAACGGAACAAATCACCACCTTT<br>CATGATGTGGAAACTCCAAATAGGATCAATACCCCCATGGCTCAAGACACTT<br>CATCGGCTCGGAGCATGGATGATACGCACAGTATTATTCAG
SBV (335nt)	TTGGAACTACGCATTCTCTGACGAGCATGTACAAGTTCAGATGGATGATAGTG<br>TAGAAAGAGTTTATGATGAAGGGAACCAAGTCTATTACTATCCGCCCCCTAA<br>ACCAGAGGGTTTTAGTTTGAACAATGTACGGACTTCGGTTAGTACGTTGTGTA<br>ATATGCTTGGAAAAGTAGTAACTCCTGAGAGAGCAATGAAAACAGCGCTGTG<br>TGCAACTCCTTATTTTGGATCAGCTTATATGACAGCTACTACCCTAGACGCTA<br>TAGGTAGTATGCAGAATACCGTTACGGGAGCTGCACACCAGTTGACTGCATC<br>TGTTGATGCGAGGTTAGAGC

microorganisms MDPI

Table S2 - Result of statistical tests comparing the levels of DWV across patrilines

N: number of patrilines that were included in the analysis (sample size ≥ 6 per patriline), Test: test used for the comparison (KW: Kruskal Wallis when >2 groups compared, if significant, Dunn test corrected with Bonferroni to compare patrilines pairwise; W: Wilcoxon–Mann–Whitney tests to compare two groups). All groups compared are composed by at least 6 samples.

<table>
<tr><th>Group</th><th>N (Test)</th><th>DWV-A</th><th>DWV-B</th></tr>
<tr><td>Col B (I)</td><td>3 (KW)</td><td>d = 2.6169, df = 3, p = 0.45</td><td>d = 0.1709, df = 3, p= 0.98</td></tr>
<tr><td>Col D (II)</td><td>3 (KW)</td><td>d = 7.7302, p = 0.02
<table>
<tr><th></th><th>G</th><th>J</th></tr>
<tr><td>J</td><td>1.812
p = 0.105</td><td>-</td></tr>
<tr><td>K</td><td>2.732
p = 0.009*</td><td>0.920
p = 0.537</td></tr>
</table></td><td>d = 8.5439, p = 0.01
<table>
<tr><th></th><th>G</th><th>J</th></tr>
<tr><td>J</td><td>-2.678
p = 0.011*</td><td>-</td></tr>
<tr><td>K</td><td>-0.325
p = 1.000</td><td>2.353
p = 0.0279</td></tr>
</table></td></tr>
<tr><td>Col E (I)</td><td>2 (W)</td><td>W = 48, p = 0.002388</td><td>W = 48, p = 0.002388</td></tr>
<tr><td>Col E (II)</td><td>2 (W)</td><td>W = 22, p = 0.8518</td><td>W = 43, p = 0.0168</td></tr>
</table>

Table S3 - Results of the Wilcoxon–Mann–Whitney tests to compare cells with reproducing and non-reproducing mites

Only groups with sample sizes ≥ 6 samples were compared.

Colony	DWV-A	DWV-B
**B**	W = 109, *p* = 0.3565	W = 120, *p* = 0.6001
**D**	W = 74.5, *p* = 0.2186	W = 81.5, *p* = 0.08741
